# Influence of ovarian reserves on assisted reproductive and perinatal outcomes in patients with endometriosis: a retrospective study

**DOI:** 10.3389/fendo.2023.1084927

**Published:** 2023-05-12

**Authors:** Shuai Liu, Yaxin Guo, Fei Li, Lei Jin

**Affiliations:** Reproductive Medicine Center, Tongji Hospital, Tongji Medical College, Huazhong University of Science and Technology, Wuhan, China

**Keywords:** endometriosis, assisted reproduction, diminished ovarian reserve, live birth rate, cumulative live birth rate, abnormal perinatal outcome

## Abstract

**Objective:**

To investigate the association between different ovarian reserves and reproductive and adverse perinatal outcomes in patients with endometriosis.

**Design:**

Retrospective study.

**Setting:**

Reproductive Medicine Center in a hospital.

**Patients:**

Patients surgically diagnosed with endometriosis were divided into three groups according to their ovarian reserve: diminished ovarian reserve (DOR) group (n=66), normal ovarian reserve (NOR) group (n=160), and high ovarian reserve (HOR) group (n=141).

**Intervention(s):**

None.

**Main Outcome Measures:**

Live birth rate (LBR), cumulative live birth rate (CLBR), and adverse perinatal outcome for singleton live births.

**Results:**

There were significantly higher live birth and cumulative live birth rates in endometriosis patients with NOR or HOR than in those with DOR. For adverse perinatal outcomes, patients with NOR or HOR had no significant association with preterm birth, gestational hypertension, placenta previa, fetal malformation, abruptio placentae, macrosomia, or low birth weight, except for a decreased risk of gestational diabetes mellitus.

**Conclusion:**

Our study revealed that although patients with endometriosis with NOR and HOR had increased reproductive outcomes, patients with endometriosis with DOR had still an acceptable live birth rate and a similar cumulative live birth rate with available oocytes. Moreover, patients with NOR and HOR might not exhibit a decreased risk of abnormal perinatal outcomes, except for gestational diabetes mellitus. Multicenter prospective studies are needed to further clarify the relationship.

## 1 Introduction

Ovarian reserve is defined as the quantity and quality of follicles within the ovary. Numerous markers, such as age, baseline antral follicle count (AFC), follicle stimulating hormone (FSH) level, and anti-Müllerian hormone (AMH) level, have been evaluated to assess ovarian reserve and predict ovarian response and reproductive potential (1).

Endometriosis is an estrogen-dependent disease in women of reproductive age that is characterized by the presence of endometrial-like tissue outside the uterine cavity (2). Endometriosis affects approximately 10% of women of reproductive age, and its prevalence among infertile women is 5–50% (3). On the one hand, patients with endometriosis often have a high risk of obstetric complications (4). A recent study found that endometriosis may adversely affect perinatal outcomes, especially due to increased risk of placenta abruption and operative delivery (5). On the other hand, the cause of infertility in women with endometriosis is multifactorial, and diminished ovarian reserve (DOR) is of major concern in women with endometriosis-associated infertility (6). Cystectomy and surgery for endometriosis, as well as the endometriomas themselves, may cause DOR. Usually, DOR results in a decreased fecundability, along with a reduction in oocyte quantity and a decrease in oocyte quality (7). Patients with DOR also have a high risk of obstetric complications. DOR, specifically defined as an AFC of six or less, is associated with a higher incidence of preeclampsia and multiple placental fetal vascular lesions (8). Study also found that younger women with low prognosis and normal ovarian reserve have a higher probability for live births and better perinatal outcomes compared with older women with poor or normal ovarian reserve (9). Nevertheless, Sunkara et al. (10) found an increased risk of adverse obstetric outcomes among women with excessive ovarian response while no increased risk among women with poor ovarian response. Whether the ovarian reserve influences perinatal outcomes in patients with endometriosis remains unclear. Therefore, it is important to determine whether there are differences in perinatal outcomes in patients with endometriosis with different ovarian reserves who become pregnant using assisted reproductive technology (ART).

In this study, we evaluated the risk of abnormal perinatal outcomes in patients with endometriosis with normal ovarian reserve (NOR) or high ovarian reserve (HOR) compared with DOR.

## 2 Materials and methods

### 2.1 Patients

We retrospectively reviewed patients who underwent their first fresh cycle of *in vitro* fertilization (IVF) or intracytoplasmic sperm injection (ICSI) between January 1, 2016 and December 12, 2019 at the Reproductive Medicine Center of Tongji Hospital, Tongji Medical College of Huazhong University of Science and Technology (Wuhan, China). This study was approved by the ethical committee of Tongji Hospital, Tongji Medical College, Huazhong University of Science and Technology (TJ-IRB20220119). Patients were surgically diagnosed with endometriosis (laparotomy or laparoscopy) and histologically confirmed from biopsies. Patients with endometrial cysts in the ovaries and adenomyosis were included in the study.

Patients were excluded from the study if they met any of the following exclusion criteria: 1) polycystic ovary syndrome; 2) endocrine diseases; 3) donated oocyte; 4) hypertension; 5) autoimmune disease.

Patients were divided into three groups by their ovarian reserve according to a clinical guideline (11): DOR group: age > 35 years or AMH < 1.1 or AFC < 5 or FSH > 12; NOR group: age ≤ 35 years and 1.1 ≤ AMH ≤ 4.5, and 7 ≤ AFC ≤14 and FSH ≤ 10; HOR group: age ≤ 35 years and AMH > 4.5 or AFC > 20.

### 2.2 Controlled ovarian stimulation protocol

The controlled ovarian stimulation(COS) protocol was individually selected according to ovarian reserve testing and other characteristics of GnRH agonist (GnRH-AGO) or antagonist (GnRH-ANTA) treatment. Human chorionic gonadotropin (hCG) (10,000 IU, EMD Serono) was used to trigger ovulation when one or two leading follicles attained a mean diameter of 18 mm. Transvaginal ultrasound-guided oocyte retrieval was conducted 34–36 h after hCG administration. The oocyte maturation rate was defined as the number of MII oocytes divided by the number of retrieved oocytes. Normal fertilization was defined as 2PN. The normal fertilization rate was defined as the number of 2PN divided by the number of retrieved oocytes in *in vitro* fertilization (IVF) or 2PN divided by the number of MII in ICSI. All embryos were checked on the morning of Day 3 after oocyte retrieval. Fewer than two embryos of the best quality were selected for transfer on Day 3.

### 2.3 Main outcome measures

We collected data on maternal age, follicle-stimulating hormone (FSH) level, body mass index (BMI), AFC, AMH level, duration of infertility, and type of endometriosis.

The COS outcomes included endometrial thickness, no. of retrieved oocytes, MII oocytes, 2PN zygotes, oocyte maturation rate, and normal fertilization rate.

The ART outcomes included biochemical pregnancy, clinical pregnancy, pregnancy loss, live birth, cumulative pregnancy, and cumulative live birth. Biochemical pregnancy was defined as a pregnancy when a woman has a positive pregnancy test, but no gestational sac can be visualized by ultrasound. Clinical pregnancy was defined as viable intrauterine pregnancy (gestational sac with fetal heart activity) confirmed by ultrasound. Clinical pregnancies and live births were calculated according to the first fresh transfer cycle. Cumulative pregnancy and live birth were calculated by the first fresh transfer cycle and subsequent frozen cycles until live birth, or all embryos were used. Women without a live birth by December 31, 2019 were considered as non-live births as a conservative estimate.

The adverse singleton perinatal outcomes included preterm birth, placenta previa, fetal malformation, gestational hypertension, gestational diabetes mellitus, low birth weight (< 2,500 g), and macrosomia (> 4,000 g).

### 2.4 Statistical analysis

Continuous data are presented as means ± standard deviations (SDs), and categorical data are presented as percentages (%). Continuous variables, with normal distribution and homogeneity of variance, were compared using the one-way analysis of variance test while continuous variables with non-normal or heterogeneity, were compared using the Kruskal–Wallis test. Categorical variables were compared using the chi-square test, Bonferroni correction was applied to all multiple comparisons, and subgroup analysis was performed with Cochran-Mantel-Haenszel test with common odds ratios calculated. The relationships among variables, subsequent clinical pregnancy, and live birth were assessed using binomial logistic regression analysis with enter method, and the odds ratio (OR) with 95% confidence intervals (CI) was calculated. A two-tailed P<0.05 was considered statistically significant. All analyses were performed using the Statistical Package for Social Sciences (SPSS version 26.0, IBM Corp, Armonk, NY, USA).

## 3 Results

### 3.1 Baseline characteristics of patients, and COS and ART outcomes

A total of 367 participants were included in the study during their first fresh cycle. When subdividing cycles according to ovarian reserve DOR (n=66), NOR (n=160), and HOR (n=141), ovarian reserve increased with decreasing age and FSH and increasing AMH levels. AFC significantly increased from DOR to NOR to HOR. The duration of infertility and BMI were comparable among the three groups. GnRH protocols differ significantly among DOR, NOR, and HOR. The detailed baseline characteristics and COS outcomes can be seen in the **Table 1**.

**Table 1:** Baseline characteristics and COS and ART outcomes.

Variable		GROUP			P value	
	DOR N=66	NOR N=160	HOR N=141	D VS N	D VS H	N VS H
Age, y	33.24 ± 4.22	29.48 ± 2.88	29.33 ± 2.77			
FSH, IU/L	8.48 ± 2.65	7.45 ± 1.51	7.01 ± 1.82			
AFC	7.77 ± 4.76	9.52 ± 2.00	15.53 ± 4.67			
AMH, ng/ml	2.49 ± 2.33	2.77 ± 0.92	7.38 ± 2.91			
BMI, kg/m^2^	20.94 ± 2.49	21.46 ± 2.50	20.95 ± 2.22	ns	ns	ns
GnRH protocol						
antagonist	28(42.4%)	20(12.5%)	9(6.4%)	<0.001	<0.001	ns
agonist	38(57.6%)	140(87.5%)	132(93.6%)	<0.001	<0.001	ns
Duration of infertility, y	3.29 ± 3.11	3.21 ± 2.10	3.02 ± 1.93	ns	ns	ns
Type of endometriosis						
endometriosis cysts	48(72.7%)	138(86.3%)	126(89.4%)	ns	ns	ns
adenomyosis	13(19.7%)	12(7.5%)	11(9.8%)	0.024	0.039	ns
cysts co-occurrence with adenomyosis	5(7.6%)	10(6.3%)	4(2.8%)	ns	ns	ns
Endometrial thickness, mm	11.76 ± 2.62	12.54 ± 2.71	12.24 ± 2.38	ns	ns	ns
No. of oocytes retrieved	8.39 ± 4.50	9.73 ± 4.48	13.94 ± 4.20	ns	<0.001	<0.001
No. of MII	7.76 ± 4.32	8.69 ± 4.24	12.20 ± 4.04	ns	<0.001	<0.001
Oocyte maturation rate	0.92 ± 0.10	0.89 ± 0.12	0.88 ± 0.13	ns	0.044	ns
No. of 2PN	5.38 ± 3.32	6.13 ± 3.42	8.40 ± 3.43	ns	<0.001	<0.001
Normal fertilization rate	0.66 ± 0.22	0.66 ± 0.21	0.64 ± 0.18	ns	ns	ns
Biochemical pregnancy	5(7.6%)	8(5.0%)	5(3.5%)	ns	ns	ns
Pregnancy loss	10(15.2%)	12(7.5)	10(7.1%)	ns	ns	ns
Clinical pregnancy	32(48.5%)	96(60%)	97(68.8%)	ns	0.015	ns
Live birth	22(33.3%)	84(52.5%)	87(61.7%)	0.027	<0.001	ns
single	17(25.8%)	59(36.9%)	66(46.8%)	ns	0.012	ns
multiple	5(7.6%)	25(15.6%)	21(14.9%)	ns	ns	ns
Cumulative pregnancy	44(66.7%)	129(80.6%)	120(85.1%)	ns	0.006	ns
Cumulative live birth	37(56.1%)	116(72.5%)	112(79.4%)	0.048	<0.001	ns
single	29(43.9%)	84(52.5%)	86(61.0%)	ns	ns	ns
multiple	8(12.1%)	32(20.0%)	26(18.4%)	ns	ns	ns

Continuous data are reported as mean ± standard deviation. Categorical data are reported as n (%). One-way analysis of variance test was used for the continuous data, and chi-square test was used for categorical data. P<0.05 was considered statistically significant. ns, not statistically significant; DOR, diminished ovarian reserve; NOR, normal ovarian reserve; HOR, high ovarian reserve; AFC, antral follicle count, FSH, follicle stimulating hormone; AMH, anti-Müllerian hormone.

Regarding the COS and ART outcomes, there were no significant differences regarding endometrial thickness, normal fertilization rate, biochemical pregnancy, and pregnancy loss among the three groups. The number of retrieved oocytes; no. of MII and no. of 2PN were significantly increased in the HOR group compared with those in the DOR and NOR groups. The oocyte maturation rate was significantly lower in the HOR group than in the DOR group (P= 0.044). Clinical pregnancy and cumulative pregnancy increased with increasing ovarian reserve from DOR to NOR to HOR and exhibited a significant difference between DOR and HOR (P=0.015), whereas live birth and cumulative live birth increased with increasing ovarian reserve from DOR to NOR to HOR with significant differences between DOR and NOR (P=0.027), DOR and HOR (P<0.001) in live birth and DOR and NOR (P=0.048), DOR and HOR (P<0.001) in cumulative live birth. The single live birth was significantly lower in DOR group than in HOR group (P=0.012). The multiple live birth, single and multiple cumulative live birth were comparable among the three groups.

Binary logistic regression analysis was performed to evaluate the effects of ovarian reserve, COS protocols, and type of endometriosis on clinical pregnancy, live births, cumulative pregnancy, and cumulative live births in women with endometriosis. The results in **Table 2** indicate that ovarian reserve was significantly associated with clinical pregnancy between HOR and DOR group (P=0.013, OR=2.276, 95%CI: 1.190~4.352) and comparable between NOR and DOR group (P>0.05). For live birth, ovarian reserve was significantly associated between NOR and DOR (P=0.031, OR=2.004 95%CI 1.068~3.763), HOR and DOR group (P=0.002, OR=2.874 95%CI 1.489~5.547). For cumulative pregnancy, ovarian reserve was significantly associated with cumulative pregnancy between HOR and DOR group (P=0.037, OR=2.225, 95%CI: 1.051~4.709) and comparable between NOR and DOR group (P>0.05), while patients with adenomyosis was significantly associated with cumulative pregnancy compared with those with endometriosis cysts (P=0.047, OR=0.463 95%CI 0.217~0.989). For cumulative live birth, ovarian reserve was significantly associated between HOR and DOR group (P=0.020, OR=2.266, 95%CI: 1.140~4.507) and comparable between NOR and DOR group(P>0.05). Other characteristics did not suggest any significant variation in the model.

**Table 2:** Binary logistic regression analysis on reproductive outcomes.

Reproductive outcome	variable	P	ORs	95% C.I.	
				Lower	Upper
Clinical pregnancy	GnRH protocol				
	antagonist		1.000		
	agonist	0.845	1.064	0.573	1.976
	ovarian reserve	0.039			
	DOR		1.000		
	NOR	0.164	1.544	0.837	2.849
	HOR	0.013	2.276	1.190	4.352
	Type of endometriosis	0.875			
	endometriosis cysts		1.000		
	adenomyosis	0.724	0.879	0.430	1.797
	co-occurrence	0.728	1.189	0.447	3.162
	Constant	0.791	0.919		
Live birth	GnRH protocol				
	antagonist		1.000		
	agonist	0.296	1.396	0.746	2.610
	ovarian reserve	0.007			
	DOR		1.000		
	NOR	0.031	2.004	1.068	3.763
	HOR	0.002	2.874	1.489	5.547
	Type of endometriosis	0.977			
	endometriosis cysts		1.000		
	adenomyosis	0.925	0.966	0.471	1.983
	co-occurrence	0.851	1.096	0.422	2.849
	Constant	0.008	0.411		
Cumulative pregnancy	GnRH protocol				
	antagonist		1.000		
	agonist	0.215	1.546	0.777	3.078
	ovarian reserve	0.111			
	DOR		1.000		
	NOR	0.148	1.664	0.835	3.315
	HOR	0.037	2.225	1.051	4.709
	Type of endometriosis	0.138			
	endometriosis cysts		1.000		
	adenomyosis	0.047	0.463	0.217	0.989
	co-occurrence	0.844	0.890	0.278	2.847
	Constant	0.068	1.860		
Cumulative live birth	GnRH protocol				
	antagonist		1.000		
	agonist	0.075	1.780	0.943	3.358
	ovarian reserve	0.065			
	DOR		1.000		
	NOR	0.144	1.609	0.850	3.045
	HOR	0.020	2.266	1.140	4.507
	Type of endometriosis	0.179			
	endometriosis cysts		1.000		
	adenomyosis	0.067	0.507	0.245	1.049
	co-occurrence	0.638	0.782	0.281	2.178
	Constant	0.826	1.073		

Odds ratios (ORs) and 95% confidence intervals (CIs) are based on the logistic regression analysis. OR and P value for ovarian reserve, GnRH protocol, and type of endometriosis using a binary logistic regression analysis for each reproductive outcome. P<0.05 was considered statistically significant. DOR, diminished ovarian reserve; NOR, normal ovarian reserve; HOR, high ovarian reserve.

**Figure 1:**
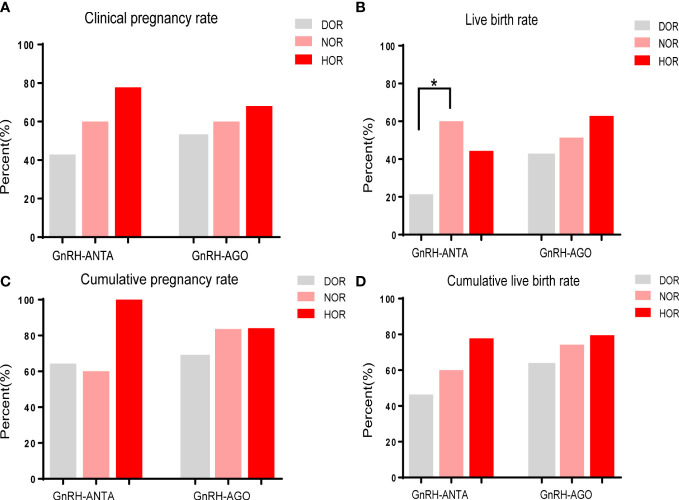
Subgroup analysis for reproductive outcomes among different ovarian reserve group by stratifying the women into GnRH-AGO and GnRH-ANTA subgroups: **(A)** Clinical pregnancy rate. **(B)** Live birth rate. **(C)** Cumulative pregnancy rate. **(D)** Cumulative live birth rate.

Subgroup analysis with Cochran-Mantel-Haenszel test was performed by stratifying the women into GnRH-AGO and GnRH-ANTA groups. **Figure 1** shows that in the GnRH-AGO group, clinical pregnancy, live birth, cumulative pregnancy, and cumulative live birth were comparable among the ovarian reserve groups. In the GnRH-ANTA group, live births were significantly lower in the DOR group (21.4%) than in the NOR group (60%), with a common odds ratio= 2.024(P=0.025) and homogeneity of the Odds Ratio(P>0.05), whereas clinical pregnancy, cumulative pregnancy, and cumulative live births were similar among the three groups.

### 3.2 Singleton perinatal outcomes

We then investigated the effect of ovarian reserve on singleton perinatal outcomes. 37 patients achieved cumulative live births with 29 singleton live births in the DOR group, among 116 patients in the NOR group, 84 had singleton live births, and among 112 patients in the HOR group, 86 had singleton live births. Singleton perinatal outcomes included abnormal outcomes are reported in **Table 3**. There were significant differences regarding gestational diabetes mellitus between DOR and NOR group (P=0.039), DOR and HOR group(P=0.015), but no significant differences were observed in the remaining outcomes.

**Table 3:** Singleton perinatal outcomes.

Variable		GROUP			P value	
	DOR N=29	NOR N=84	HOR N=86	D VS N	D VS H	N VS H
Age, y	32.72 ± 3.95	29.71 ± 2.65	29.33 ± 3.20			
FSH, IU/L	8.14 ± 2.69	7.28 ± 1.58	6.88 ± 1.48			
AFC	8.10 ± 5.13	9.56 ± 1.98	15.46 ± 4.66			
AMH, ng/mL	2.59 ± 2.63	2.73 ± 0.90	7.67 ± 3.17			
BMI, kg/m^2^	20.93 ± 2.52	21.61 ± 2.44	21.02 ± 2.18	ns	ns	ns
Type of endometriosis						
endometriosis cysts	22(75.9%)	73(86.9%)	80(93.0%)	ns	ns	ns
adenomyosis	6(20.7%)	10(11.9%)	4(4.7%)	ns	ns	ns
cysts co-occurrence with adenomyosis	1(3.4%)	1(1.2%)	2(2.3%)	ns	ns	ns
Gestational age, d	271.83 ± 8.89	270.46 ± 12.23	271.28 ± 9.44	ns	ns	ns
Birth weight, g	3356.55 ± 403.96	3281.67 ± 513.29	3283.20 ± 483.24	ns	ns	ns
Delivery mode						
Natural labor	23(79.3%)	66(78.6%)	58(67.4%)	ns	ns	ns
Cesarean delivery	6(20.7%)	18(21.4%)	28(32.6%)	ns	ns	ns
Gender						
Male	18(62.1%)	45(53.6%)	45(52.3%)	ns	ns	ns
Female	11(37.9%)	39(46.4%)	41(47.7%)	ns	ns	ns
Abnormal perinatal outcomes						
Preterm birth < 37 week	2(6.9%)	9(10.7%)	9(10.5%)	ns	ns	ns
Gestational hypertension	0	3(3.6%)	4(4.7%)	ns	ns	ns
Gestational diabetes mellitus	7(24.1%)	6(7.1%)	5(5.8%)	0.039	0.015	ns
Placenta previa	4(13.8%)	8(9.5%)	5(5.8%)	ns	ns	ns
Fetal malformation	0	0	4(4.7%)	ns	ns	ns
Abruptio placentae	0	3(3.6%)	0	ns	ns	ns
Macrosomia > 4,000 g	2(6.9%)	3(3.6%)	4(4.7%)	ns	ns	ns
Low birth weight < 2,500 g	0	7(8.3%)	4(4.7%)	ns	ns	ns

Continuous data are reported as mean ± standard deviation. Categorical data are reported as n (%). One-way analysis of variance test was used for the continuous data, and chi-square test was used for categorical data. P<0.05 was considered statistically significant. ns, not statistically significant; DOR, diminished ovarian reserve; NOR, normal ovarian reserve; HOR, high ovarian reserve; AFC, antral follicle count, FSH, follicle stimulating hormone; AMH, anti-Müllerian hormone.

For abnormal perinatal outcome, binary logistic regression analysis was conducted for ovarian reserve, type of endometriosis, and COS protocol. The correlation was that ovarian reserve had a significant association with the risk of gestational diabetes mellitus, and the NOR (7.1%) (OR: 0.199, 95% CI: 0.057–0.697, P=0.012) and HOR (5.8%) (OR: 0.151, 95% CI: 0.040–0.568, P=0.005) groups had a decreased risk of gestational diabetes mellitus when compared with the DOR group (24.1%) (OR: 1.000). No association was observed with the remaining abnormal perinatal outcomes. The risks of low birth weight, macrosomia, preterm birth, gestational hypertension, placenta previa, fetal malformation, and abruptio placentae were similar among the three groups.

## 4 Discussion

This study aimed to explore the relationship between reproductive and perinatal outcomes and ovarian reserve in patients with endometriosis. The results showed that patients with endometriosis with NOR and HOR had increased reproductive outcomes compared to patients with DOR, but the reproductive outcomes in patients with DOR were still acceptable. Regarding adverse perinatal outcomes, patients with NOR and HOR had a decreased risk of gestational diabetes mellitus compared to patients with DOR. Patients with DOR and HOR did not exhibit a higher risk of abnormal perinatal outcomes in the remaining outcomes.

Ovarian reserve testing, which is closely associated with reproductive outcomes, is a useful option for physicians to assess ovarian reserve. Diminished ovarian reserve is defined as decreased oocyte quality, quantity, or reproductive potential, resulting in infertility (12). In our study, we found that the reproductive outcomes were increased in patients with endometriosis with NOR or HOR than in those with DOR. Nevertheless, patients with endometriosis with DOR could still have an acceptable live birth rate (33.3%) and a cumulative live birth rate (56.1%) with available oocytes. Moreover, live birth was significantly increased in patients with NOR than in those with DOR choosing GnRH-ANTA, and no significant difference was found in clinical pregnancy, cumulative clinical pregnancy, and cumulative live birth among patients with different ovarian reserve with GnRH-ANTA or GnRH-AGO. This result indicated that in fresh embryo transfer cycles, patients with endometriosis with NOR would have a better live birth rate than those with DOR, and with sufficient available oocytes, these patients could reach a similar cumulative live birth rate in subsequent frozen cycles. A previous study also revealed that for patients with endometriosis with DOR, the GnRH-AGO protocol may achieve better clinical IVF-ET outcomes (13). However, another study reported that when combined with the frozen-embryo transfer strategy, the GnRH-ANTA protocol had comparable clinical pregnancy outcomes as the GnRH-AGO protocol in patients with DOR. Cohen et al. (14) found that women with diminished ovarian reserves have low live birth rates after the first IVF-ICSI cycle. Yu Deng et al. (15) also found no statistically significant differences in the cumulative live birth rate in women with DOR and endometriosis. Van Rooij et al. (16) reported that when patients were defined as poor responders based on the number of AFC, the results of the comparison of poor and normal responders were similar because they could also have oocytes available. In our COS outcomes, we found that the oocyte maturation rate and normal fertilization rate were similar between the DOR and NOR groups, indicating that patients with endometriosis with DOR might still have available oocytes. Finally, with similar percentages of available oocytes, endometriosis patients with DOR achieved an acceptable cumulative live birth rate.

Another clinically relevant finding is the singleton perinatal outcome. In a recent study, women with endometriosis had an increased risk of adverse perinatal outcomes compared to those with other reproductive diseases, including miscarriage, preeclampsia, gestational hypertension, preterm deliveries, placenta previa, and caesarean section (17–21). A previous study found that women with high ovarian response had a higher risk of adverse obstetric outcomes of preterm deliveries and low birth weight (10). However, our study found that the risks of preterm birth, gestational hypertension, placenta previa, fetal malformation, abruptio placentae, macrosomia, and low birth weight did not differ significantly between patients with endometriosis with NOR or HOR and those with DOR. The risk of gestational diabetes mellitus was extremely low in the NOR and HOR group than DOR group. One possible explanation is that women with infertility may be at greater risk for gestational diabetes mellitus overall, and the risk increases with age (22, 23). Patients in the NOR and HOR group were younger, which might therefore exhibit decreased risk of developing gestational diabetes mellitus than DOR group. Therefore, we found that endometriosis is associated with DOR, affecting quantity, but not embryo quality, and would not impact subsequent abnormal perinatal outcomes.

To our knowledge, this is the first study to evaluate abnormal perinatal outcomes in patients with endometriosis with different ovarian reserves. Our study has several limitations. As a retrospective study, although we corrected for several known confounders, the potential for unrecognized confounders still remains. Due to the more frequent application of GnRH-AGO protocol than GnRH-ANTA protocol in infertility females with endometriosis in China, and the strict inclusion and exclusion criteria, the sample size in the GnRH-ANTA group was relatively small, further prospective study with large sample size was necessary. Moreover, our results were based only on patients in their first fresh cycle, the effect of frozen-thawed cycle on perinatal outcomes need further investigation.

In conclusion, our study revealed that although patients with endometriosis with NOR and HOR had increased reproductive outcomes, patients with endometriosis with DOR had still an acceptable live birth rate and a similar cumulative live birth rate with available oocytes. Moreover, patients with NOR and HOR might not exhibit a decreased risk of abnormal perinatal outcomes, except for gestational diabetes mellitus. Multicenter prospective studies are needed to further clarify the relationship.

## Data availability statement

The raw data supporting the conclusions of this article will be made available by the authors, without undue reservation.

## Ethics statement

The studies involving human participants were reviewed and approved by the ethical committee of Tongji Hospital, Tongji Medical College, Huazhong University of Science and Technology. Written informed consent for participation was not required for this study in accordance with the national legislation and the institutional requirements.

## Author contributions

LJ and FL conceived of and designed the study. SL and YG collected the data. SL analyzed the data and wrote the paper. All authors contributed to the article and approved the submitted version.
